# Ischemic Heart Disease Hospitalization among Older People in a Subtropical City — Hong Kong: Does Winter Have a Greater Impact than Summer?

**DOI:** 10.3390/ijerph110403845

**Published:** 2014-04-04

**Authors:** Pui Hing Chau, Moses Wong, Jean Woo

**Affiliations:** 1 School of Nursing, The University of Hong Kong, Pokfulam, Hong Kong; 2 Department of Medicine & Therapeutics, The Chinese University of Hong Kong, Clinical Sciences Building, Prince of Wales Hospital, Shatin, N.T., Hong Kong; E-Mails: moseswong@cuhk.edu.hk (M.W.); jeanwoowong@cuhk.edu.hk (J.W.)

**Keywords:** excess winter morbidity, hospitalization, cold weather, subtropical climate, Hong Kong, older population, ischemic heart disease

## Abstract

Globally, excess winter morbidity from ischemic heart disease (IHD) is reported. In subtropical regions, there is a need to quantify the difference in the adverse effect of cold winters compared with hot summers, particularly among the older people. Our objectives were to: (i) compare the effect of winter on IHD hospitalizations with that of summer; (ii) examine temporal trends in the excess winter hospitalizations; and (iii) investigate the effect of age, gender, and meteorological factors on predicting such excess. Inpatient admissions due to IHD as principal cause during June 2000 to February 2009 in public hospitals of Hong Kong were extracted for the population aged ≥65. An Excess Hospitalization in Winter *vs.* Summer (EHWS) Index was used to contrast the adverse effect of weather on hospitalizations in winter *vs.* summer. Multiple linear regressions were used to investigate the trend and the predictors of such index. It was found that in a subtropical city, greater effect of winter on IHD hospitalizations than summer was observed, particularly among the oldest old (an index of 61.5% (95% CI: 49.5%–74.4%) for men aged ≥85 and 32.3% (95% CI: 25.5%–39.5%) for women aged ≥85). There was significant increasing trend in the index among those aged ≥85 but the age difference was less prominent among the women. Absolute level of coldness was not a significant factor, whereas the change in temperature was a significant factor, which implies that great fluctuation in temperature within a winter day had greater impact on occurrence of circulatory disease than an absolute temperature threshold.

## 1. Introduction

Aging is related to impaired homeostasis in response to environmental stressors. One such stressor is environmental temperature [1]. There is extensive literature on excess winter morbidity and mortality worldwide, including regions in both Northern and Southern hemispheres [2,3,4,5,6,7]. It has been documented that the circulatory system is more sensitive to weather stress. Higher blood pressure and increased coronary thrombosis have been shown to be associated with lower environmental temperature [8,9]. However this phenomenon is not only observable in cold winters, and it could also be observed in hot summers [10]. There is a need to quantify the difference in impact of winter on the occurrence of circulatory diseases as compare to summer, particularly in a subtropical region.

Hong Kong is located at the southeastern coast of China which has a subtropical climate, where summer is hot and humid, and winter is cold and dry. During winter, the normal monthly means of daily minimum temperature ranges from 14.5 °C in January to 15.9 °C in December [11]. Normally, the average number of days with daily minimum temperature of 12 °C or below ranges from 3.3 days in December to 6.8 days in January [12]. However, adverse effects due to occasional extreme cold temperature are still observable. Excess cardiovascular mortality in winter was observed among the Hong Kong population, particularly the older population [13,14,15]. In a previous study, we also found excess IHD hospitalizations in winter in both community-dwelling and institutionalized older people, with more prominent effect among the institutional population [16].

A different optimal temperature range may apply to the subtropical region. It has been documented that the effect of cold weather on cardiovascular mortality was more prominent than the effect of heat in countries where the weather is predominantly warm and hot [17]. In a study conducted in Shanghai, China, cold weather was shown to exert a higher impact on cardiovascular hospital admissions than hot weather [18]. This would likely be the case in Hong Kong where the population is used to be exposed to warmer climate. Temperature-wise, even though the local winter is milder and less stressful than those countries located in the temperate zone of the world, the effect of temperature drop from sudden and persistent cold spells, in the Hong Kong context, can already lead to adverse health effect.

The Hong Kong population is aging rapidly. The proportion of people aged 65 and above will reach 30% by year 2041 [19]. If the older population is more prone to the adverse effect of cold weather, the increasing number of older people would result in more IHD hospitalizations in future winters. There is a need to examine the excess IHD hospitalizations in winter, explore possible reasons, and formulate recommendations to the health service providers and the public. Since adverse health effects of hot weather have also been reported in Hong Kong [14,15], we will contrast the effect of cold winter to hot summer. The objectives of this study were to: (i) compare the effect of winter on IHD hospitalizations with that of summer; (ii) examine temporal trends in excess IHD hospitalizations in winter; and (iii) investigate the relationship between such excess hospitalizations with age group, gender, and meteorological factors. 

## 2. Methods

### 2.1. Data Collection

Hospital discharge data for the Hong Kong population aged 65 years and above were obtained from the Clinical Management System database of the Hong Kong Hospital Authority (HA). This inpatient dataset contains information on patients’ age, gender, principal diagnosis of admission, date of admission and discharge for each admission. The dataset covers discharges during 2000–2009; admissions with discharge date after 31 December 2009 were not included. Admissions due to IHD were identified by the principal admission diagnosis, which was coded according to the International Classification of Diseases (9th Revision, Clinical Modification [ICD-9-CM]) as codes 410–414. This dataset represents the majority of IHD inpatients in Hong Kong, since 88%–94% of IHD admissions were made to public hospitals (www.ha.org.hk). To distinguish new IHD episodes from inter-hospital transfers and re-admissions, hospital admissions within 30 days from the last date of discharge were classified as the same episode.

Meteorological data were principally recorded and obtained from the Hong Kong Observatory (www.hko.gov.hk), including: (i) number of cold days; (ii) number of very hot days; (iii) number of hot nights; (iv) number of hours with Cold Weather Warning; (v) number of hours with Very Hot Weather Warning; (vi) daily minimum temperature; (vii) daily maximum temperature; (viii) daily mean temperature; (ix) daily mean relative humidity; (x) daily mean wind speed; and (xi) number of hours with reduced visibility. Meteorological data (i) to (v) were based on the general situation of the whole territory taking into account measurements at various monitoring stations. Meteorological data (vi) to (x) were based on the measurements taken at the monitoring station located at the Hong Kong Observatory’s Headquarters at Tsim Sha Tsui, which is often used as the reference for the local weather. Daily data were used to calculate the total number in the season or the average number during the season. No missing value was observed from the data. The Hong Kong Observatory defines cold days as days with daily minimum temperature ≤12 °C, very hot days as days with daily maximum temperature ≥33 °C, and hot nights as days with daily minimum temperature ≥28 °C. Temperature falling into the range for defining cold days, very hot days, and hot nights is considered as extreme temperature in Hong Kong. Corresponding weather warnings are issued by the Hong Kong Observatory whenever Hong Kong experiences cold and very hot weather, to inform and advise public to take appropriate precautionary actions to the danger of temperature-induced adverse health effects. Local literature showed that the issue of Very Hot Weather Warning had protective effect towards IHD and stroke mortality among the older population [20]. Thus, the number of hours with Very Hot Weather Warning and Cold Weather Warning were included. Reduced visibility has been used as a proxy to air pollution and was shown to be associated with increased cardiovascular mortality among the older Hong Kong population [21]. Thus, the number of hours of reduced visibility, defined by the Hong Kong Observatory as visibility below 8 km, with relative humidity below 95%, no fog, mist, or precipitation, was used as a proxy for air pollution. Net Effective Temperature (NET) is a well-defined measure to summarize the combined effect of ambient temperature, relative humidity and wind speed [22]. The Hong Kong Observatory found that a NET above 26 °C or below 14 °C was likely to increase mortality rates, particularly among the older population [14]. Hence, we calculated the daily NET based on the formula using temperature, humidity and wind speed as variables [14,22], and counted the number of days with NET above 26 °C and number of days with NET below 14 °C for the analysis. There were studies showing that larger daily temperature range, defined as daily maximum temperature minus daily minimum temperature, was related to higher hospitalization [23,24,25,26]. Hence, the mean daily temperature ranges in summer and in winter were calculated for analysis. There were also studies about the effect of daily temperature on health [13,23,24]. Hence, mean daily minimum temperature in winter, mean daily maximum temperature in summer, mean daily mean temperature in winter and mean daily mean temperature in summer were also calculated for examination.

Ethics approval was obtained from The University of Hong Kong and The Chinese University of Hong Kong.

### 2.2. Data Analysis

Hospitalization episodes in winter were defined by the date of admission during the coldest months of the year, *i.e.*, December of the year, and January to February in the following year, and summer from June to August. Hence, with data available for years 2000 to 2009, only nine complete winters (ending February 2008) could be used for data analysis. Excess Hospitalization in Winter *vs.* Summer (EHWS) Index was developed to assess the effects of cold weather on hospitalization by difference in episodes between winter and summer months, in a similar manner as the commonly used Excess Winter Mortality Index [27,28,29]. Similar to the Excess Winter Mortality Index, the EHWS Index adjusted for the difference in number of calendar days in winter *vs.* summer, with an aim to contrast the number of hospitalization in different seasons, despite the annual variation in level of coldness or warmness. While the duration of the cold period might affect the number of adverse health events, there are other factors, such as level of coldness or other unobserved conditions, which might be difficult to adjust for. Therefore, only the number of calendar days was adjusted as a proxy to the winter weather and summer weather. EHWS was defined by:

EHWS = *n_w_* − (*n_S_* × 90/92)

where *n_w_* is the number of hospitalization episodes in winter and *n_S_* is the number of hospitalization episodes in summer. The constant (90/92) is for adjusting the difference in number of days in the winter and summer months. In leap years, the constant (91/92) was used. The EHWS Index, expressed as percentage, was defined by:

EHWS Index (I) = EHWS/(*n_S_* × 90/92)

A positive index indicated that there were higher than expected hospitalizations in winter compared to those in summer, and *vice versa*. A zero index indicated equal hospitalizations in winter and those in summer, adjusted for the duration of the season. To reflect the precision in the estimation of the index, a Confidence Interval (CI) was constructed. A CI covering zero implied an insignificant difference between the number of hospitalization in winter and in summer. The calculation of the confidence limits was based on the methods suggested in existing reference [28]. Similar to other ratio estimates, such as relative risks and odds ratios, the natural logarithm of the ratio approximately follows Normal distribution [30]. Therefore, the confidence limits for a ratio were usually given by taking exponential of the confidence limits of the natural logarithm of the ratio. Furthermore, the formula of the confidence limits had to take into account that the EHWS Index involved subtraction of the hospitalizations in summer (which is also the denominator) in the numerator. Hence, the 95% CI of EHWS Index were calculated by:

Lower confidence limit = [(I + 1)/exp(1.96 × sqrt (1/n_w_ + 1/n_S_))] − 1

Upper confidence limit = [(I + 1) × exp(1.96 × sqrt (1/n_w_ + 1/n_S_))] − 1

To examine the trend in IHD EHWS Index, multiple linear regression was used with the annual IHD EHWS Index in 2000 to 2008 as the dependent variable. Age groups (65–74, 75–84, ≥85), gender, and year were used as independent variables. The interactions between the independent variables were included to examine if the trends were different across different age groups and gender. Insignificant interaction terms (*p* > 0.05) were removed from the model.

To examine the effects of meteorological factors on the IHD EHWS Index, multiple linear regression was used with the annual IHD EHWS Index as the dependent variable and age groups and gender were entered as independent variables. The meteorological variables, including (i) number of cold days in winter; (ii) number of very hot days in summer; (iii) number of hot nights in summer; (iv) number of hours with Cold Weather Warning in winter; and (v) number of hours with Very Hot Weather Warning in summer; (vi) number of days with Net Effective Temperature (NET) below 14 °C in winter; (vii) number of days with NET above 26 °C in summer; (viii) mean daily temperature range in winter; (ix) mean daily temperature range in summer; (x) mean daily minimum temperature in winter; (xi) mean daily maximum temperature in summer; (xii) mean daily mean temperature in winter; (xiii) mean daily mean temperature in summer; (xiv) number of hours with reduced visibility in winter; and (xv) number of hours with reduced visibility in summer, were used as independent variables, the significant predictors would be selected to the final model using the stepwise procedure (entry *p* < 0.05 and removal *p* > 0.10). The residuals from the final model were examined for the regression model assumptions (linearity, independence, homoscedasticity, and normality). Absence of multicollinearity was checked by a variance inflation factor (VIF) of less than 5.

SPSS version 20 (SPSS Inc. Chicago, IL, USA) was used for all the statistical analyses. A significant level of 5% was adopted.

## 3. Results

Table 1 shows a summary of the meteorological data in Hong Kong during June 2000 to February 2009. There were on average 14.4 cold days, 10.4 very hot days and 17.1 hot nights each year. On average, there were 68.1 days with NET below 14 °C each year and 20.3 days with NET above 26 °C. Mean daily range of temperature was similar in winter and in summer (4 °C). Daily mean temperature varied from 17.4 °C in winter to 28.4 °C in summer. The average daily minimum temperature in winter was 15.5 °C, whereas the average daily maximum temperature in summer was 30.8 °C.

**Table 1:** Descriptive statistics of the meteorological data in Hong Kong, June 2000 to February 2008.

Variables	Mean	Range
Number of cold days in winter	14.4	5−32
Number of very hot days in summer	10.4	3−25
Number of hot nights in summer	17.1	8−22
Number of hours with Cold Weather Warning in winter	414.7	216.8−834.6
Number of hours with Very Hot Weather Warning in summer	257.5	126.0−415.8
Number of days with NET below 14 °C in winter	68.1	63−78
Number of days with NET above 26 °C in summer	20.3	12−26
Number of hours with reduced visibility in winter	427.2	248–593
Number of hours with reduced visibility in summer	82.2	19–217
Daily mean temperature in winter (in °C)	17.4	8.2−24.3
Daily mean temperature in summer (in °C)	28.4	22.5−31.8
Daily temperature range in winter (in °C)	4.1	0.7−12.1
Daily temperature range in summer (in °C)	4.3	0.9−9.8
Daily minimum temperature in winter (in °C)	15.5	6.4−23.3
Daily maximum temperature in summer (in °C)	30.8	23.2−35.4

A total of 54,869 IHD hospitalization episodes made in summers and winters during June 2000 to February 2009 were identified for the analysis, with 29,536 episodes from men and 25,333 from women. Admissions during winter months accounted for 29,435 episodes (equivalent to an average of 36.3 episodes per day), whereas 25,434 admissions were made during summer (equivalent to an average of 30.7 episodes per day). Accounting for the different number of days in the two seasons, there were an excess of 4492 admissions (18.0%) in winter as compared to summer. Excess admissions due to IHD during winter were found, irrespective of age group and gender (Table 2). Mean EHWS Index due to IHD hospitalizations exacerbated with advanced age for both gender. The greatest gender difference in EHWS Index was found at age above 85.

**Table 2:** Mean IHD EHWS Index (in %) among the older population in Hong Kong, by age group and gender, 2000–2008.

Gender	Age Group	IHD EHWS Index (in %)	95% Confidence Interval (in %)
Male	65–74	7.5	(4.2, 10.9)
	75–84	27.1	(22.4, 32.0)
	≥85	61.5	(49.5, 74.4)
Female	65–74	4.7	(0.5, 9.1)
	75–84	21.0	(16.5, 25.7)
	≥85	32.3	(25.5, 39.5)

**Figure 1:**
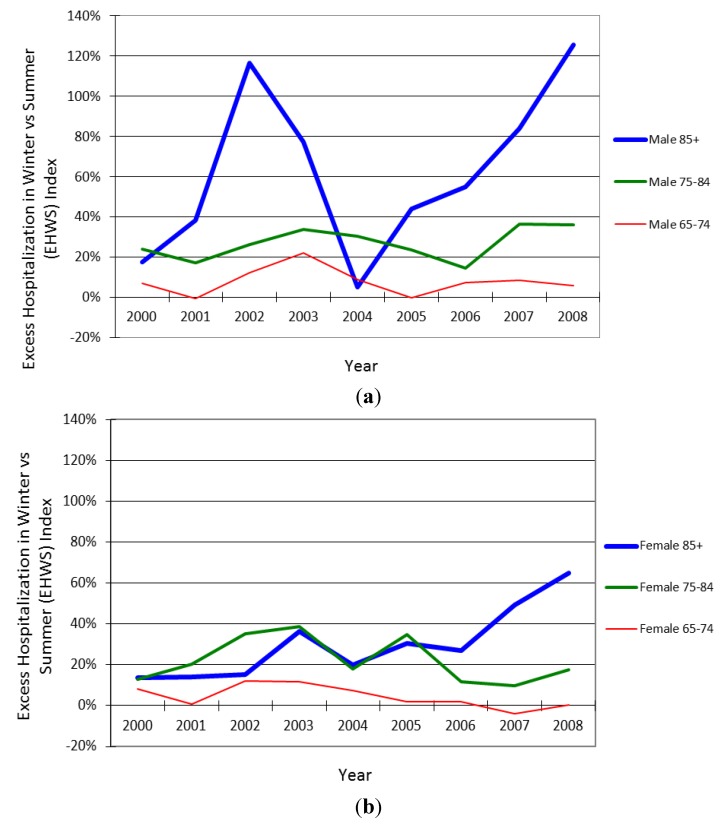
(**a**) IHD EHWS Index among the male older population in Hong Kong, by age group, 2000–2008; (**b**) IHD EHWS Index among the female older population in Hong Kong, by age group, 2000–2008.

Figure 1 shows that all age and gender groups had a positive EHWS Index, implying excess winter hospitalization, except for men aged 65–74 in 2001 and 2005 and women aged 65–74 in 2007 where slightly more summer admissions were observed.

For the trend analyses, interaction terms between gender and year were insignificant (*p* = 0.399), implying the trends in IHD EHWS Indices were indifferent between the two gender. Meanwhile, interaction terms between age group and year were significant (*p* = 0.007), implying the trends in IHD EHWS Indices differed by age groups. After removing insignificant interaction terms between gender and year, the final multiple linear regression model showed that there was significant increasing trend in IHD EHWS Index among those aged 85 and above (*p* < 0.001) but not among those younger age groups. The model also showed that IHD EHWS Index was significantly higher among men than women (*p* < 0.001), higher among those aged 85 and above (*p* < 0.001), and the gender difference was smaller among the younger age groups (*p* = 0.027). To investigate the observed trends in the EHWS Index, trend analyses of the hospitalization rate in winter and that in summer were performed separately. It was found that for the hospitalization rate in winter, the main effect year (*p* = 0.005), the interaction terms between gender and year (*p* = 0.008), and the interaction terms between age group and year (*p* < 0.001) were significant, indicating there was significant trends in the hospitalization rate in winter and the trends were different among the age groups and gender. The population aged 85 and above was the only age group that showed a significant trend in the hospitalization rate in winter, with an annual increase of 23.3 episodes per 100,000 population (95% CI: 15.3–31.3). Men would have an additional increase of 11.1 episodes per 100,000 population (95% CI: 3.1–19.1) per year. For the hospitalization rate in summer, there was insignificant trend (*p* = 0.091), regardless of the different age-gender subgroups.

In the regression analyses of the IHD EHWS Index on the meteorological variables, the final model based on stepwise selection procedure included mean daily temperature range in winter as significant predictor, in addition to age group and gender. Table 3 shows the fitted regression coefficients of the final model. The final model had an R-square of 0.558. Scatter plot of observed EHWS Index *vs.* predicted values showed that the linearity assumption was met by the oldest old age group, while the younger age groups had larger deviations. To examine the independence assumption, the Durbin-Watson statistic was found to be 1.5, which did not have sufficient evidence for significant residual autocorrelation at lag 1. Scatter plot of residuals *vs.* predicted value showed that homoscedasticity assumption was met for most of the values, except for the extremely large values. Normality assumption was met as shown by the test of normality of the residuals by Kolmogorov-Smirnov test. All VIF were less than 5, indicating the absence of multicollinearity. Similar to the model examining trends, this model showed that a higher IHD EHWS Index was significantly associated with older age groups, and the age difference was more prominent among men than women. It was found that when the mean daily temperature range in winter increased by 1 °C, the IHD EHWS Index would increase by 13.6 percentage points (95% CI: 1.2–26.1), implying that a larger fluctuation of temperature within a day was associated with more IHD hospitalizations in winter.

**Table 3:** Results of the final multiple linear regression model for the IHD EHWS Index (in %) among the older population in Hong Kong.

Parameter	Fitted Regression Coefficient	95% Confidence Interval
Intercept	−48.0	(−100.5, 4.5)
Age group		
65–74	0	—
75–84	19.0	(1.1, 36.9)
≥85	54.7	(36.8, 72.6)
Gender		
Female	−3.3	(−21.2, 14.6)
Male	0	—
Interaction between age group and gender		
Female 65–74	0	—
Female 75–84	−1.3	(−26.7, 24.0)
Female ≥85	−29.0	(−54.4, −3.7)
Male 65–74	0	—
Male 75–84	0	—
Male ≥85	0	—
Mean daily temperature range in winter	13.6	(1.2, 26.1)

## 4. Discussion

This study examined the adverse effect of winter on IHD hospitalizations among the Hong Kong older population aged 65 and above, using inpatient data during 2000–2009. An EHWS Index was used to contrast the adverse effect in winter *vs.* summer. The positive EHWS Index showed that there were more IHD hospitalizations in winter than in summer, suggesting that the adverse impact of winter on circulatory disease was greater than that of summer. Multiple linear regression models showed that oldest old population (aged 85 or above) had higher EHWS Index than those aged 65–84, suggesting greater impact of cold among the oldest old. A significant increasing trend in IHD EHWS Index was observed among those aged 85 and above but not the other age groups, but the trends were indifferent between the two gender. Winters with larger fluctuation of temperature within a day were associated with higher IHD EHWS Index, due to the increased winter hospitalizations.

Given an average level of daily temperature range in winter at 4.1 °C in Hong Kong in 2000–2008, the fitted regression model predicted all population subgroups except those aged 65–74 had a positive EHWS Index significantly different from zero. The population aged 85 and above had an index of 62.4 (95% CI: 49.8–75.1) for the men and 30.1 (95% CI: 17.4–42.8) for the women. Men aged 75–84 were predicted to have an index of 26.7 (95% CI: 14.1–39.4) and women of the same age group an index of 22.1 (95% CI: 9.4–34.8). For those aged 65–74, the index for men was predicted to be 7.8 (95% CI: −4.9–20.4), whilst that for women was 4.4 (95% CI: −8.2–17.1). The largest positive index among the oldest old implied that they were the mostly prone to the cold weather in winter, when different age groups were exposed to the same weather conditions. Therefore, preventive measures should be targeted at this group of the population which is growing rapidly. By year 2041, about 5.8% of the population will be classified as oldest old [19]. Furthermore, it was found that the hospitalization rate of IHD in winter was increasing among those aged 85 and above, in contrast to the static trend in summer. This further highlights the need to tailor public health strategies to avoid IHD hospitalization in winter among this high risk group.

The findings are consistent with previous findings that there were more IHD hospitalizations in winter than in summer [17,18], and that the oldest old were more sensitive to the effect of cold weather [31]. Older people are more vulnerable to cold weather than younger people since they have decreased peripheral resistance and reduced thermogenesis, which result in diminished thermoregulation of body temperature [1]. Moreover, sarcopenia and the lack of body fat among older people also contribute greatly to the diminished insulation effect from skin, muscle and fat [1,32]. The older population has additional risk to adverse health outcome from cold weather due to pre-existing diseases which affect their body temperature [31].

Mean daily temperature range in winter, which was found as risk factor to IHD EHWS Index, was increasing over the year 2000–2008 (results not shown). However, only those aged 85 or above also showed an increasing trend in the EHWS Index and IHD hospitalization in winter, implying the oldest old group was more sensitive to the fluctuation in daily temperature than the younger age groups. Furthermore, variables related to the level of coldness, such as minimum temperature, mean temperature, number of cold days, number of hours with Cold Weather Warning, and number of days having NET below 14 °C, were found to be insignificant factors. The findings implied that great fluctuation in temperature within a day had greater impact on occurrence of circulatory disease than an absolute temperature threshold. Lower body temperature, resulted from lower environmental temperature, was shown to be associated with increased platelet count, blood viscosity, plasma cholesterol concentration, and blood pressure, which might explain the rapid increases in coronary thrombosis in cold weather [8]. Furthermore, lower ambient temperature was associated with elevated blood pressure [9]. The effect of tremendous temperature change within a day might be explained by the fact that a decreased ambient temperature was associated with increased inflammatory blood markers, representing a higher risk for cardiovascular events [33]. The systolic blood pressure of older people increased after experiencing a mild cold, this increase was found to be persistent even after rewarming [32].

During the cold days, the Hong Kong government will remind the public, particularly the older people and those with chronic illnesses, to take precautionary measures, including wearing appropriately warm clothing, having sufficient energy intake, performing regular exercises, staying in warm environment, and seeking medical care if needed [34]. However, older people might not follow these advices effectively. There is no indoor heating in Hong Kong, and buildings are not built to retain heat. Energy inefficient housing which leads to low indoor temperature had been shown to relate to increased winter hospitalizations [5]. In Hong Kong, most of the air-conditioners only have cooling function, but not a warming function. The portable heaters used in domestic households, if available, are very ineffective in that it can only raise the room temperature within a very small area. People have to be very close enough to the heater to be able to feel the warmth. In institutional setting, electric wall bars are the only commonly used indoor heating equipment and the windows are kept open to allow ventilation. The indoor temperature of the institutional care facilitates was found to be below the World Health Organization’s recommendation of 18 °C [35]. Secondly, under-nutrition and being lean could affect thermoregulation among the older people [32,36]. Poor social and health conditions leading to under-nutrition are not uncommon among older people, resulting in inadequate body fat storage and insufficient calories to support thermoregulations during the cold days. Lack of physical activities was also a risk factor of cardiovascular disease [37]. Older people in Hong Kong are used to have morning exercises in parks. However, they do not go outside during cold days, which further deprive their opportunity from having enough exercise to keep circulation and warm.

Hong Kong is located in the subtropical region, the average minimum daily temperature was usually above 12 °C in winter during 2000–2008 [38], where the winter temperature is not as low as those countries. However, the effect of winter on IHD hospital admission was still observed. It could be possible that people, particularly older people, had adapted to a relatively warm climate, such that in winter, larger temperature fluctuation within a day will trigger hospital admissions due to IHD [39]. 

## 5. Strength and Limitations

The strength of this study laid on the use of a territory-wide database spanning a decade to examine the effect of cold weather on IHD hospital admissions in Hong Kong. Nevertheless, our dataset did not capture all IHD inpatients in Hong Kong, since some patients were admitted to private hospitals. Our dataset covered about 88% to 93% of all IHD hospital admissions (www.ha.org.hk). By using the EHWS Index, we examined the ratio of IHD inpatient episodes in winter to those in summer. Hence, assuming the proportion of patients utilizing the public sector was constant within a year, the influence from the patients admitted to the private hospitals should be negligible. This study only focused on inpatient episodes, but did not capture milder cases (e.g., those not requiring inpatient care) and more severe cases (e.g., fatal cases before hospitalization). Other datasets were required in order to assess any similarities or differences among different levels of IHD severity. The limitation of our study was mainly due to the observational nature. While this study only focused on the effect of weather, there might be effects from other factors such as financial stress or individual’s medical history. In addition, by using a multiple linear regression, we could only perform an exploratory analysis on the research question. While the daily temperature fluctuation partly explained the greater impact of winter on excess IHD hospitalization, much variation in the EHWS Index was left unexplained, as shown from the residual diagnostics. Other factors and mechanisms warrant further exploration and sophisticated modelling based on primary data is needed.

## 6. Suggestions

Since the older people might have difficulties in implementing preventive measures as suggested, merely raising awareness towards the cold weather in winter seems to have limited effect. Proactive strategies have to be taken to help older people prepare for the cold weather. First, a warm indoor environment with stable temperature has to be created and maintained, to avoid indoor temperature fluctuating greatly with the outdoor temperature. As recommended by the World Health Organization, the ideal indoor temperature should be kept at 18 and 2–3 °C warmer for the older people [40]. Installation of air-conditioning with warming function, or at least more efficient portable heater, is needed. Electricity subsidies to the older people so that they will be willing to use these apparatus; Second, meals on wheels have to be provided for older people who are unable to cook hot meals themselves. In addition to hot meals provided at community centers, special hot meals delivery services should be considered and enhanced in cold days, when some older people might not be willing/able to visit those centers; Thirdly, public education about indoor exercises should be promoted such that the older people can continue their regular exercises in indoor areas like home, corridor or lobby; Last but not least, everyone should pay special care and attention to the older people around them. A phone call/visit to the older people would help remind them to implement the necessary preventive measures, which would also allow to check and see if the older people are doing well.

These suggested measures should be evaluated for their effectiveness in preventing excess hospitalizations in winter. Future study can also investigate whether similar results will be found using mortality data among the same population. Moreover, epidemiological study incorporating risk factors at individual level can be conducted to further investigate the factors to the adverse health outcomes during winter.

## 7. Conclusions

This study is a large ecological study to look at IHD hospitalizations among the older population in Hong Kong. An EHWS Index was developed to contrast the adverse effect of weather on hospitalizations in winter *vs.* summer. We found that even for a subtropical city, the effect of winter on IHD hospital admission was still observable among the older population, particularly among the oldest old. Recommendations on preventive measures from the public health perspectives were made so as to minimize the adverse effect of winter.
